# Differential Trafficking of Oxidized LDL and Oxidized LDL Immune Complexes in Macrophages: Impact on Oxidative Stress

**DOI:** 10.1371/journal.pone.0012534

**Published:** 2010-09-02

**Authors:** Mohammed M. Al Gadban, Kent J. Smith, Farzan Soodavar, Christabelle Piansay, Charlyne Chassereau, Waleed O. Twal, Richard L. Klein, Gabriel Virella, Maria F. Lopes-Virella, Samar M. Hammad

**Affiliations:** 1 Department of Regenerative Medicine and Cell Biology, Medical University of South Carolina, Charleston, South Carolina, United States of America; 2 Summer Undergraduate Research Program, College of Graduate Studies, Medical University of South Carolina, Charleston, South Carolina, United States of America; 3 Division of Endocrinology, Department of Medicine, Medical University of South Carolina, Charleston, South Carolina, United States of America; 4 Research Service, Ralph H. Johnson Veterans Affairs Medical Center, Charleston, South Carolina, United States of America; 5 Department of Microbiology and Immunology, Medical University of South Carolina, Charleston, South Carolina, United States of America; University Paris Sud, France

## Abstract

**Background:**

Oxidized low-density lipoproteins (oxLDL) and oxLDL-containing immune complexes (oxLDL-IC) contribute to formation of lipid-laden macrophages (foam cells). It has been shown that oxLDL-IC are considerably more efficient than oxLDL in induction of foam cell formation, inflammatory cytokines secretion, and cell survival promotion. Whereas oxLDL is taken up by several scavenger receptors, oxLDL-IC are predominantly internalized through the FCγ receptor I (FCγ RI). This study examined differences in intracellular trafficking of lipid and apolipoprotein moieties of oxLDL and oxLDL-IC and the impact on oxidative stress.

**Methodology/Findings:**

Fluorescently labeled lipid and protein moieties of oxLDL co-localized within endosomal and lysosomal compartments in U937 human monocytic cells. In contrast, the lipid moiety of oxLDL-IC was detected in the endosomal compartment, whereas its apolipoprotein moiety advanced to the lysosomal compartment. Cells treated with oxLDL-IC prior to oxLDL demonstrated co-localization of internalized lipid moieties from both oxLDL and oxLDL-IC in the endosomal compartment. This sequential treatment likely inhibited oxLDL lipid moieties from trafficking to the lysosomal compartment. In RAW 264.7 macrophages, oxLDL-IC but not oxLDL induced GFP-tagged heat shock protein 70 (HSP70) and HSP70B', which co-localized with the lipid moiety of oxLDL-IC in the endosomal compartment. This suggests that HSP70 family members might prevent the degradation of the internalized lipid moiety of oxLDL-IC by delaying its advancement to the lysosome. The data also showed that mitochondrial membrane potential was decreased and generation of reactive oxygen and nitrogen species was increased in U937 cell treated with oxLDL compared to oxLDL-IC.

**Conclusions/Significance:**

Findings suggest that lipid and apolipoprotein moieties of oxLDL-IC traffic to separate cellular compartments, and that HSP70/70B' might sequester the lipid moiety of oxLDL-IC in the endosomal compartment. This mechanism could ultimately influence macrophage function and survival. Furthermore, oxLDL-IC might regulate the intracellular trafficking of free oxLDL possibly through the induction of HSP70/70B'.

## Introduction

An early event in atherosclerosis is the engorgement of macrophages with lipids. It is well established that activated macrophages become lipid-laden foam cells by taking up oxidatively modified low-density lipoprotein (oxLDL), leading to the accumulation of cholesteryl esters (CE) [1]. Circulating oxLDL elicits the production of auto-immune antibodies, predominantly of the pro-inflammatory IgG1 and IgG3 isotypes, resulting in the formation of oxLDL-containing immune complexes (oxLDL-IC) [2], [3], [4]. While both oxLDL and oxLDL-IC have been detected in human atherosclerotic plaques [5], oxLDL-IC are considerably more efficient than oxLDL in the induction of foam cell formation [6]. We and others have shown that human monocytic cells exposed to oxLDL have reduced cell survival compared to those exposed to oxLDL-IC [7], [8]. Furthermore macrophages exposed to oxLDL-IC result in the release of the pro-inflammatory and plaque destabilizing factors that promote lesion progression [9], [10], [11].

The internalization of lipids in macrophages occurs through mechanisms involving different cell surface receptors. The macrophage scavenger receptors are a family of proteins which include scavenger receptors class A (macrophage scavenger receptor I and II, MSR-I and MSR-II), and class B (SR-BI and CD36). Macrophage scavenger receptors from both classes bind modified LDL [12], [13], and mediate its delivery to lysosomes for processing and degradation [14]. In contrast, oxLDL-IC are predominantly internalized through the FCγ receptor I (FCγ RI) [15]. However, the temporal and spatial intracellular localization of lipid and apolipoprotein moieties of oxLDL-IC, and how trafficking of these moieties influences the formation, activation and survival of foam cells are still obscure. In a recent study we showed that in macrophages, internalized oxLDL-IC induces a member of the HSP70 family, heat shock protein 70B' (HSP70B'), which co-localizes with the lipid moiety of oxLDL-IC [16]. In the current study we investigated the effect of HSP70 and HSP70B' on the advancement of internalized moieties of oxLDL-IC to the lysosomal compartment.

Based on experimental evidence and clinical studies, oxidative and nitrosative stresses have been shown to be induced by atherosclerosis risk factors and to contribute to the onset and development of atherosclerotic vascular damage [17]. Reactive oxygen species (ROS), as well reactive nitrogen species (RNS), are products of normal cellular metabolism; however, cells of the immune system produce both superoxide anion (O_2_^−^) and nitric oxide (NO) during the oxidative burst triggered during inflammatory processes [18]. The dynamic interactions between endogenous ROS/RNS and intracellular signaling pathways may play a key role in the activation of macrophages. It has been found that the generation of ROS and RNS does not completely deplete intracellular antioxidants, rather regulates the atherogenic process by modulating intracellular signaling pathways affecting inflammatory cell adhesion, migration, proliferation, and differentiation [19]. Nonetheless, overproduction of ROS/RNS or a deficiency of enzymatic or non-enzymatic antioxidants may cause biological damage to cellular lipids, proteins, and DNA leading to cell death [20].

Mitochondria are both major source and target of oxidative stress [21], [22]. It has been suggested that mitochondrial damage in early stage can predict ROS- and RNS-mediated atherosclerotic lesions [23]. However, the differential effect of oxLDL and oxLDL-IC on oxidative stress in macrophages is not fully understood. In this study we investigated differences in trafficking of internalized oxLDL and oxLDL-IC using fluorescently labeled lipoprotein moieties. We also examined the effects of oxLDL and oxLDL-IC on mitochondrial membrane potential and the intracellular generation of hydrogen peroxide (H_2_O_2_) and NO.

## Results

### Characterization of labeled oxLDL and uptake by U937 cells

**Figure 1:**
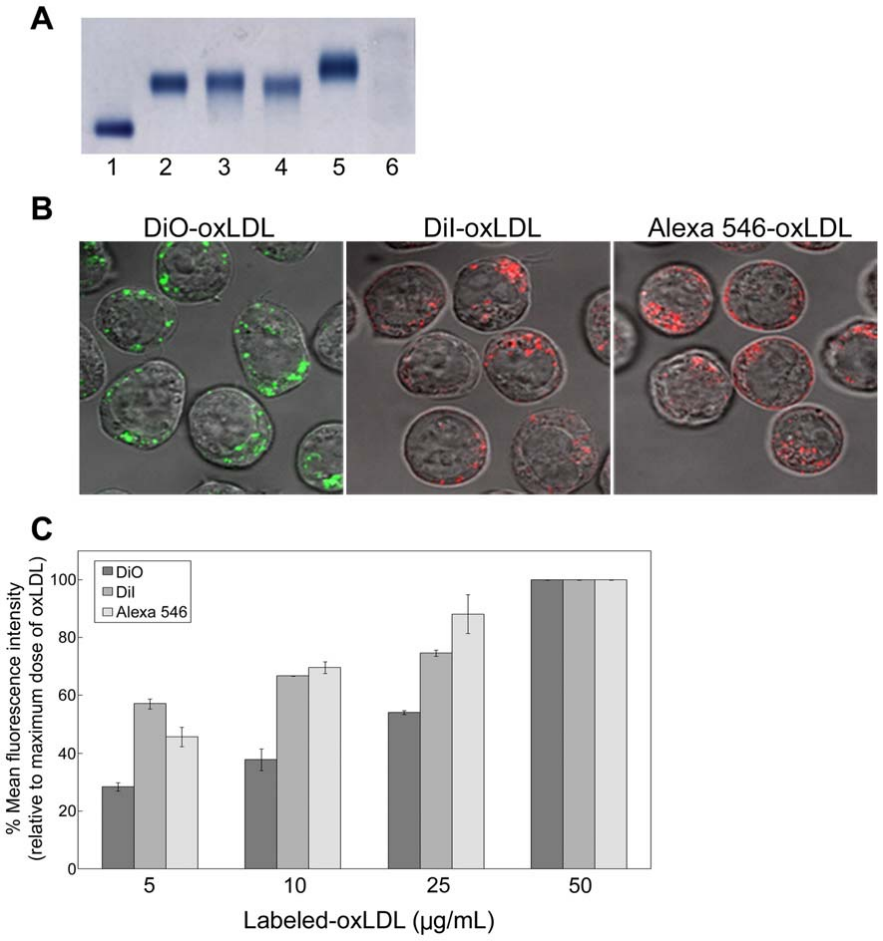
Characterization of oxLDL labeling and uptake by U937 cells. (**A**) Migration of fluorescently labeled oxLDL analyzed by agarose gel electrophoresis: lane 1: native LDL, lane 2: oxLDL, lane 3: DiI-oxLDL, lane 4: DiO-oxLDL, lane 5: Alexa 546-oxLDL, lane 6: LPDS. (**B**) Uptake of fluorescently labeled oxLDL. U937 cells were treated with labeled oxLDL: DiO-oxLDL, DiI-oxLDL, or Alexa 546-oxLDL (24 µg/ml) for 5 h then fixed with 4% formaldehyde and visualized in sealed capillaries using confocal microscopy. (**C**) FACS analysis showing dose-dependent uptake of labeled oxLDL 90 min post treatment in U937 cells. Each data point represents the mean ± range of duplicate determinations (1×10^4^ cells/determination), and data presented are representative of four independent experiments.

Oxidation of N-LDL modifies the lipoprotein particle and affects its migration on agarose gel. Paragon Lipo Gel electrophoresis system was used to verify particle modification of unlabeled as well as labeled oxLDL. Figure 1A shows the unlabeled oxLDL (lane 2) and oxLDL labeled with DiI (lane 3), DiO (lane 4), and Alexa 546 (lane 5) migrated further toward the positive pole than N-LDL (lane 1).

To visualize the uptake of labeled oxLDL, U937 cells were incubated with DiO-oxLDL, DiI-oxLDL, or Alexa 546-oxLDL for 5 h. Figure 1B shows that oxLDL labeled with any of the three fluorescent labels was internalized in U937 cells, and that labeling of either lipid or protein moiety of oxLDL resulted in comparable internalization of oxLDL. To quantify internalization of labeled lipid and protein moiety of oxLDL in a dose-dependent manner, FACS analysis was performed. A mean of 82% of the cells showed measurable levels of internalized labeled oxLDL. Figure 1C shows that the mean fluorescence intensity in response to DiO-oxLDL, DiI-oxLDL, and Alexa 546-oxLDL was dose dependent.

### Localization of labeled lipoprotein moieties of oxLDL and oxLDL-IC in endosomal compartment

**Figure 2:**
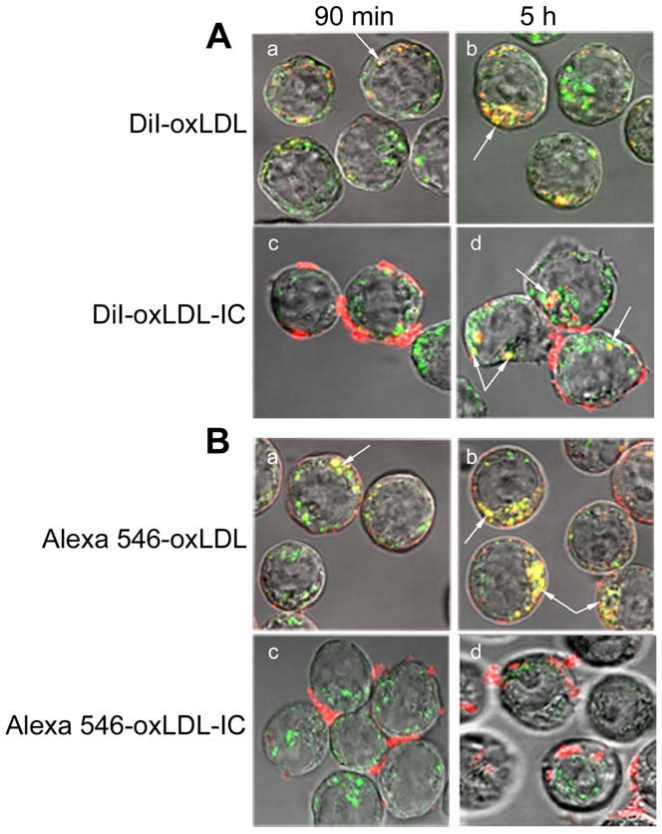
Localization of labeled lipoprotein moieties of oxLDL and oxLDL-IC in endosomal vesicles. U937 cells were treated with endosomal marker (Alexa 488-transferrin, green) and with either DiI-labeled lipid moiety (red) (**A**), or Alexa 546-labeled protein moiety (red) (**B**) for 90 min and 5 h. Cells were treated with Alexa 488-transferrin (5 µg/ml) and with either labeled oxLDL (24 µg/ml) or labeled oxLDL-IC (32 µg/ml), fixed with 4% formaldehyde, suspended in sealed capillaries and visualized using Zeiss LSM 510 laser scanning confocal microscope. Arrows point at co-localization of lipid and apolipoprotein moities of oxLDL and oxLDL-IC with Alexa 488-transferrin.

To investigate localization of internalized labeled lipoprotein moieties (lipids and apolipoproteins) of oxLDL and oxLDL-IC in the endosomal compartment, transferrin labeled with Alexa 488 was used as a marker for the early endosomal compartment. Transferrin is typically transported into the cell through endosomal vesicles, which recycle back to the cell surface. Cells were incubated simultaneously with labeled transferrin, and with either labeled oxLDL or oxLDL-IC. Cells treated with DiI-oxLDL (Fig. 2A, panels a,b) or Alexa 546-oxLDL (Fig. 2B, panels a,b), showed co-localization of lipid and apolipoprotein moieties of oxLDL with transferrin at 90 min, with an increase in uptake and co-localization with transferrin (endosomal vescicles) at 5 h. For cells treated with labeled oxLDL-IC, both the labeled lipid moiety (Fig. 2A, panel c) and apolipoprotein moiety (Fig. 2B, panel c) of oxLDL-IC were found associated with the cell membrane at 90 min. Interestingly, at 5 h post treatment, labeled lipid and apolipoprotein moieties of oxLDL-IC showed separate pathways. While internalized lipid moiety of oxLDL-IC (DiI) co-localized with transferrin (Fig. 2A, panel d); the internalized apolipoprotein moiety did not (Fig. 2B, panel d).

### Localization of labeled lipoprotein moieties of oxLDL and oxLDL-IC in lysosomal compartment

**Figure 3:**
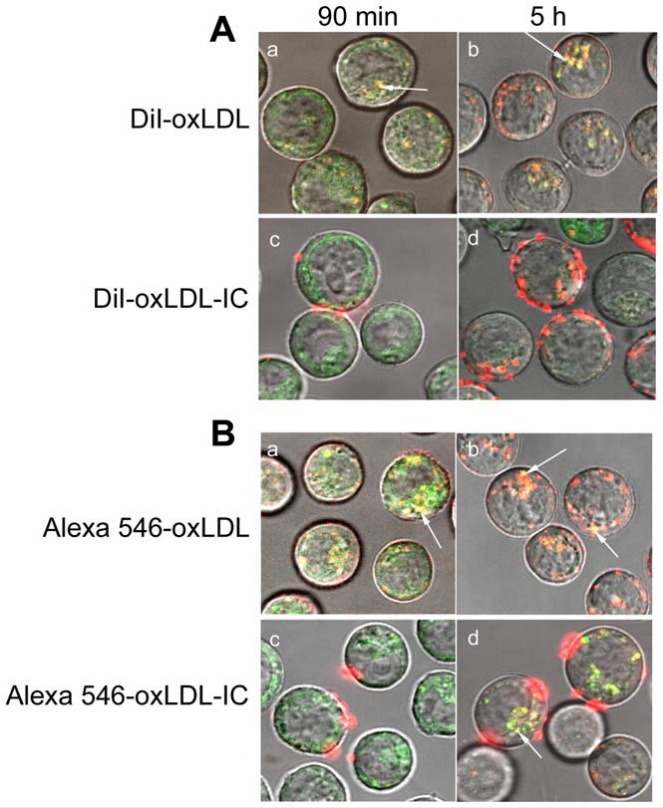
Localization of labeled lipoprotein moieties of oxLDL and oxLDL-IC in lysosomal compartment. U937 cells were treated with either DiI-labeled lipid moiety (red) (**A**), or Alexa 546-labeled protein moiety (red) (**B**) for 90 min and 5 h, with lysosomal marker (Lyso Tracker Green DND-26, 50 nM) applied for the last 30 min of incubation. Cells were treated with labeled oxLDL and oxLDL-IC at 18 µg/ml and 24 µg/ml, respectively. Live cells were washed with DPBS then suspended in sealed capillaries and visualized using confocal microscopy. Arrows point at co-localization of lipid and apolipoprotein moieties of oxLDL and oxLDL-IC with lysosomal compartment.

To determine whether oxLDL and oxLDL-IC trafficked to the lysosomal compartment, Lyso Tracker-26 was used. This fluorescent dye accumulates in cellular compartments with low internal organelle pH and can be used to investigate the biosynthesis and pathogenesis of lysosomes in live cells. For cells treated with labeled oxLDL, both the lipid (Fig. 3A; panels a,b) and apolipoprotein moieties (Fig. 3B; panels a,b) co-localized with Lyso Tracker-26 at 90 min, with an increase of oxLDL internalization at 5 h. For cells treated with labeled oxLDL-IC, labeled lipid moiety (Fig. 3A, panel c) and apolipoprotein moiety (Fig. 3B, panel c) of oxLDL-IC were found associated with the cell membrane of living U937 cells at 90 min. However, at 5 h post treatment, the internalized lipid and apolipoprotein moieties of oxLDL-IC trafficked separately. While internalized apolipoprotein moiety co-localized with Lyso Tracker-26 (Fig. 3B, panel d); the internalized lipid moiety of oxLDL-IC (DiI) did not appear to localize in the lysosomal compartment (Fig. 3A, panel d).

### Lipid moieties of oxLDL and oxLDL-IC co-localize when administered sequentially but not simultaneously

**Figure 4:**
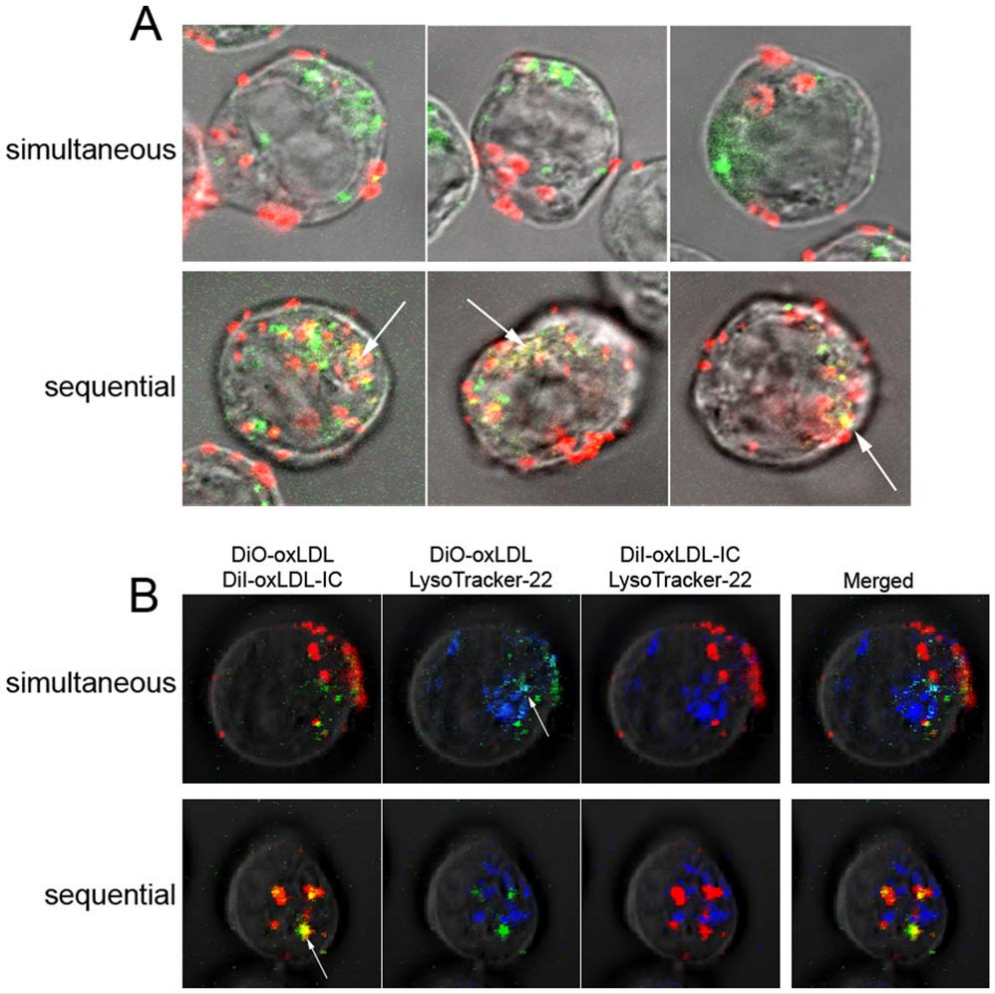
Lipid moieties of oxLDL and oxLDL-IC co-localize when administered sequentially but not simultaneously. U937 cells were incubated with DiI-oxLDL-IC (red) and DiO-oxLDL (green) (**A**) sequentially and (**B**) in parallel in U937 cells. They were incubated for a total of 5 h. Sequential experiment involved 2 h incubation of oxLDL-IC (32 µg/ml), prior to addition of oxLDL (24 µg/ml) for 3 h. Lyso Tacker Blue DND-22, (50 nM) was used and applied for the last 30 min of incubation. Cells were fixed with 4% formaldehyde, suspended in sealed capillaries and visualized using Zeiss LSM 510 Laser scanning confocal microscope. Arrows point at co-localization of oxLDL in the lysosomal compartment in the upper panel, and the co-localization of oxLDL and oxLDL-IC in the lower panel.

To determine the effect of co-incubation of oxLDL and oxLDL-IC on lipoprotein uptake and trafficking, U937 cells were treated simultaneously or sequentially with DiI-oxLDL-IC preceding DiO-oxLDL treatment. Figure 4A shows that when labeled oxLDL-IC and oxLDL were administered simultaneously their lipid moieties did not appear to co-localize 5 h post treatment. However, when administered sequentially with DiI-oxLDL-IC 2 h prior to DiO-oxLDL treatment, the internalized lipid moieties of oxLDL and oxLDL-IC co-localized. To investigate which labeled lipoprotein moieties of oxLDL and oxLDL-IC trafficked to the lysosomal compartment, Lyso Tracker-22 was used to probe the lysosomes. Figure 4B shows that after simultaneous incubation with labeled oxLDL and oxLDL-IC (upper panel), the internalized lipid moiety of oxLDL (DiO) co-localized with Lyostracker-22; however, the internalized lipid moiety of oxLDL-IC (DiI) did not appear to co-localize with either DiO or Lyso Tracker-22. Interestingly, when labeled oxLDL-IC and oxLDL were added sequentially (Fig. 4B, lower panels); the internalized lipid moieties of oxLDL (DiO) and oxLDL-IC (DiI) were co-localized, but not with the Lyso Tracker-22.

### Lipid moiety of oxLDL-IC but not oxLDL co-localizes with induced HSP70/70B' in endosomal vesicles

**Figure 5:**
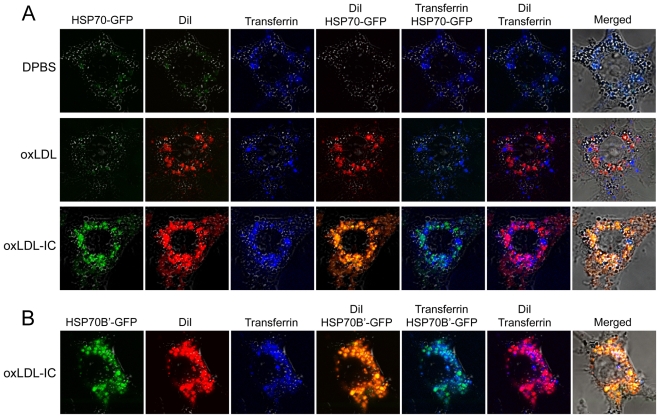
Lipid moiety of oxLDL-IC but not oxLDL co-localizes with induced HSP70/70B' in endosomal vesicles. RAW 264.7 cells were transfected with HSP70-GFP (**A**) or HSP70B'-GFP (**B**), then treated with DiI-oxLDL (24 µg/ml), DiI-oxLDL-IC (32 µg/ml), or DPBS vehicle in serum-free DMEM for 3 h. Alexa 633-transferrin (10 µg/ml) was then added for an additional 2 h. Cells were then fixed with 4% formaldehyde, washed with DPBS, and visualized using confocal microscopy.

We have recently shown that in RAW 267.4 cells both HSP70-GFP and HSP70B'-GFP are induced by oxLDL-IC but not oxLDL [16]. Here we show that DiI-oxLDL-IC co-localized with Alexa 633-transferrin and HSP70-GFP (Fig. 5A) or HSP70B'-GFP (Fig. 5B). RAW 267.4 cells treated with DiI-oxLDL showed co-localization of the lipid moiety (DiI) of oxLDL with labeled transferrin in HSP70-GFP- transfected cells (Fig. 5A) or HSP70B'-GFP-transfected cells (data not shown).

### Differential effect of oxLDL and oxLDL-IC on mitochondrial membrane potential and ROS/RNS production

**Figure 6:**
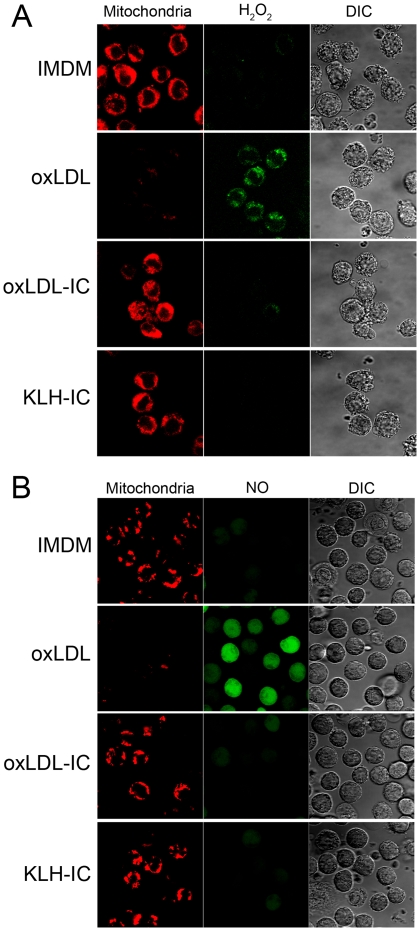
Differential effect of oxLDL and oxLDL-IC on mitochondrial membrane potential and ROS/RNS production. U937 cells were grown in phenol red-free IMDM supplemented with 10% fetal bovine serum, 100 units/ml penicillin, 50 µg/ml streptomycin, and IFN-γ (200 ng/ml) for 18 h then treated with oxLDL (90 µg/ml), oxLDL-IC, KLH-IC (120 µg/ml) in IMDM up to 5 h. (**A**) Detection of H_2_O_2_: Cells were treated with CM-H2DCFDA (5 µM), and Mito Tracker (100 nM) for 30 and 15 min, respectively, prior to conclusion of incubation time with the treatments (5 h). Cells were fixed, suspended in sealed capillaries and visualized using Zeiss LSM 510 Laser scanning confocal microscope. (**B**) Detection of NO: IFN-γ-treated cells were incubated with L-arginine (100 µM), and DAF-FM diacetate (10 µM) for 1 h, then washed with IMDM twice. Cells were then treated with oxLDL, oxLDL-IC, and KLH-IC as indicated above. Mito Tracker (100 nM) were added 15 min prior to conclusion of incubation time with the treatments (90 min). Live cells were washed, suspended in sealed capillaries and visualized using Zeiss LSM 510 Laser scanning confocal microscope.

Mitochondrial membrane potential and generation of ROS and RNS were examined using MitoTracker® Deep Red FM, CM-H2DCFDA and DAF-FM diacetate, respectively. Mitochondrial membrane potential was decreased in cells treated with oxLDL compared to cells treated with oxLDL-IC, KLH-IC, or IMDM vehicle (Fig. 6A,B). Cells treated with oxLDL-IC, KLH-IC, or IMDM vehicle maintained mitochondrial membrane potential at all time points (data not shown). Additionally oxLDL induced corresponding increase in H_2_O_2_ levels (Fig. 6A) and NO levels (Fig. 6B). The generation of H_2_O_2_ and NO in response to oxLDL occurred as early as 30 min post treatment (data not shown) and increased over time. There was no difference in H_2_O_2_ and NO generation in response to oxLDL-IC, KLH-IC or IMDM vehicle at all time points (data not shown).

## Discussion

Investigating the mechanisms controlling the intracellular transport of lipid and apolipoprotein moieties of oxLDL and oxLDL-IC in macrophages is central in understanding foam cell formation, activation, and survival. In this study we examined the uptake of oxLDL and oxLDL-IC by human monocytic cells and analyzed differences in the intracellular trafficking of their lipid and apolipoprotein moieties. We showed that both lipid and protein moieties of oxLDL were localized in endosomal and lysosomal compartments; however, the internalized lipid and apolipoprotein moieties of oxLDL-IC trafficked separately. The internalized lipid moiety of oxLDL-IC localized in the endosomal vesicles, whereas the apolipoprotein moiety advanced to the lysosmal compartment. Cells treated with oxLDL-IC prior to addition of oxLDL demonstrated co-localization of internalized lipid moieties from both oxLDL and oxLDL-IC. This sequential treatment with oxLDL-IC followed by oxLDL likely inhibited oxLDL lipid moieties from trafficking to the lysosomal compartment, which suggests that oxLDL-IC might regulate intracellular trafficking of free oxLDL.

Intriguingly, the lipid moiety of oxLDL-IC, but not oxLDL, co-localized in the endosomal compartment with induced HSP70/70B'. This suggests that HSP70 family members might prevent the degradation of the internalized lipid moiety of oxLDL-IC by delaying its advancement to the lysosome. The results also showed that mitochondrial membrane potential was decreased, and the generation of reactive oxygen and nitrogen species was increased in monocytic cells treated with oxLDL compared to oxLDL-IC and control immune complexes. It has been previously shown that constitutive and inducible HSP70s contribute to oxidative resistance evoked by heat shock or ethanol [24]. The current data provide further mechanistic support to our argument that oxLDL-IC are considerably more efficient than oxLDL in induction of lipid accumulation [3], [6], [25], and in promotion of cell survival [7].

Modified forms of LDL, including oxLDL and oxLDL-IC, have been detected in circulation and in atheromatous plaques [5], [26], and have been shown to contribute to macrophage lipid accumulation in the vessel wall [5], [26], [27], [28]. The accumulation of cholesterol in macrophages in the form of cytoplasmic lipid droplets is an early step in the formation of atherosclerotic lesions [28]. Continual macrophage uptake of oxLDL increases the accumulation of CE, resulting in lysosomal disturbance and ultimately apoptosis [29], [30]. In the present study we corroborated that the uptake of both lipid and protein moieties of oxLDL was dose dependent in U937 cells, and that both lipid and protein moieties of oxLDL were internalized in endosomal vesicles and localized in the lysosomal compartment.

Although the oxLDL used to prepare oxLDL-IC was the same preparation as that of the free oxLDL, the trafficking of the lipid and protein moieties of oxLDL was different when the lipoprotein presented to cells free or as a part of an immune complex. Oxidized LDL, when free, binds to a number of cell-surface receptors, including scavenger receptors SR-A and CD36 which are the critical contributors to modified lipoprotein uptake in macrophages [1], [31], [32]. In contrast, oxLDL-IC are predominantly internalized through the Fcγ RI [15], resulting in an increased release of cytokines and CE accumulation when compared to cells exposed to oxLDL alone [3], [25]. The data presented in this study showed that both lipid and apolipoprotein moieties of oxLDL-IC were still associated with the cell membrane of U937 cells 90 min post treatment. After 5 h the lipid and apolipoprotein moieties of oxLDL-IC were internalized, however they took separate paths inside the cells. Interestingly, the lipid moiety of oxLDL-IC localized in the endosomal vesicles, while the apolipoprotein moiety trafficked directly to the lysosomal compartment. These results suggest that the internalized lipid moiety of oxLDL-IC may have been “trapped” in the endosomal vesicles, whereas the apolipoprotein moiety of oxLDL-IC was rapidly directed to the lysosomal compartment for degradation.

It has been previously shown that human macrophages incubated with LDL-IC 22 h prior to addition of native LDL for another 20 h resulted in increase in intracellular accumulation of “undegraded” LDL, and 90% of the accumulated CE were attributed to the initial incubation with LDL-IC [33]. Here, we showed that when cells were incubated with oxLDL-IC and oxLDL simultaneously, their internalized lipid moieties did not appear to co-localize; however, when cells were treated with oxLDL-IC for 2 h prior to addition of oxLDL, internalized lipid moieties of both oxLDL and oxLDL-IC co-localized. This sequential incubation interfered with the lipid moiety of oxLDL trafficking to the lysosomal compartment. Typically and as demonstrated in this study, free modified lipoproteins internalized by macrophages are swiftly trafficked to lysosomes [34]. Simultaneous incubation of oxLDL with oxLDL-IC resulted in trafficking of the lipid moiety of oxLDL to the lysosmal compartment with almost no interference of oxLDL-IC.

These results suggest that stimulation with oxLDL-IC may regulate the trafficking and degradation of internalized oxLDL possibly through generating a signaling cascade to redirect the lipid moiety of free oxLDL away from lysosomes. Alternatively, the delay or prevention of trafficking to the lysosomal compartment of lipoprotein constituents may be attributed to chaperon binding and/or entry into intracellular vesicular recycling compartments destined for the cell surface. It has been shown for example that in dendritic cells antigens endocytosed by Fcγ RIIB access a non-degenerative intracellular vesicular compartment that recycles to the cell surface facilitating antigen presentation to lymphocytes [35]. Since HSPs are molecular chaperones that bind protein and non-protein molecules with exposed hydrophobic residues [36], HSP70/70B' could possibly be involved in the “sequestration” of lipoprotein constituents in the endosomal compartment as shown in the current study. Interestingly, it has been recently shown that endocytosed recombinant HSP70 stabilizes lysosomes by binding to an endolysosomal anionic phospholipid bis(monoacylglycero)phosphate, an essential co-factor for lysosomal sphingomyelin metabolism [37].

In macrophages and dendritic cells, it has been also shown that complexes formed by binding of the lipid-binding protein, β_2_-glycoprotein I (β_2_GPI), to oxLDL or liposomes containing phospholipids, facilitate processing and presentation of a cryptic β_2_GPI's epitope to autoreactive T cells from antiphospholipid syndrome patients [38]. Interestingly, in the presence of anti-β_2_GPI IgG, β_2_GPI-oxLDL complexes were shown to be rapidly incorporated into lysosomes, while the non-complexed β_2_GPI stagnated in late endosomes [39]. The expression of the receptors CD36 and FCγ RI were up-regulated in response to β_2_GPI-oxLDL complexes in combination with anti-β_2_GPI IgG. Efficient presentation of the cryptic determinants by monocytes was found to be mediated by FCγ RI [40].

It has been shown that excess production of ROS and RNS has been implicated with vascular lesion formation and functional defect [41], [42], [43]. It is now clear that H_2_O_2_ and NO produced by the respiratory burst function as second messengers and activates major signaling pathways [18]. For example, H_2_O_2_ activates both the nuclear factor-κB and activator protein-1 transcription factors, both control the inducible expression of genes regulating inflammatory responses [44]. Our present data demonstrated that, in cells treated with free oxLDL, mitochondrial membrane potential decreased and intracellular H_2_O_2_ and NO significantly increased compared to cells treated with oxLDL-IC or KLH-IC. Our data are in agreement with data by Deng et al [45], who showed that differentiated U937 cells treated with oxLDL exhibited mitochondrial membrane depolarization and increased H_2_O_2_ and NO levels by 15 min post treatment. Here we additionally show that cells exposed to oxLDL-IC maintained unvarying mitochondrial membrane potential, and exhibited no change in their baseline levels of H_2_O_2_ and NO over time.

In an earlier study we examined the effects of oxLDL compared to oxLDL-IC on global gene expression in U937 cells using microarray analysis [46]. The data revealed that levels of expression of genes encoding superoxide dismutase 2 *(SOD2)* was up-regulated 71-fold in response to oxLDL-IC compared to vehicle. SOD2 is a principal scavenger enzyme in mitochondrial matrix, which protects cells from oxidative stress by detoxifying superoxide generated in mitochondrial respiration by dismutation. In contrast, heme oxygenase 1 *(HMOX1)* was the gene with the greatest level of increase in response to oxLDL [46]. HMOX1 induction is known to lead to an increase in catalytic free iron release and it has been suggested that HMOX1 expression can be increased several fold by stimuli that induce cellular oxidative stress, including oxidized LDL [47], [48]. Our present data demonstrated that cells treated with oxLDL exhibited reduced mitochondrial membrane potential and increased H_2_O_2_ levels compared to oxLDL-IC, KLH-IC immune complexes. Cells treated with immune complexes maintained unvarying mitochondrial membrane potential. These results suggest that, in human macrophages, free oxLDL and oxLDL-IC could be involved in the differential regulation of mitochondrial respiratory chain function and possibly influencing foam cell activation and survival.

In conclusion our current data showed that internalized lipid and apolipoprotein moieties of oxLDL-IC trafficked to separate cellular compartments, and that oxLDL-IC can regulate the trafficking and metabolism of internalized free oxLDL. The results also showed that cells treated with oxLDL and oxLDL-IC regulated mitochondrial membrane potential and H_2_O_2_ production differentially. Our current findings may contribute to future development of therapeutics targeting certain aspects of lipoprotein trafficking in macrophages and foam cells.

## Materials and Methods

### Cells

The human monocytic cell line U937 was obtained from the American Type Culture Collection (Manassas, VA) (ATCC CRL-1593.2). This line is a monocytic lymphoma cell line, which originates from resident macrophages [49]. Cells were maintained in Iscove's modified Dulbecco's medium (IMDM) supplemented with 10% fetal bovine serum, 100 units/ml penicillin, and 50 µg/ml streptomycin at 37°C, 5% CO_2_. Unless otherwise indicated, cells were seeded at 2.5×10^5^ cells/250 µl medium in 48-well plates, and incubated in serum-free medium in the presence of interferon-gamma (IFN-γ) (200 ng/ml) (EMD, Bioscience, Sandiego, CA) for 18 h prior to addition of experimental treatments. RAW 264.7 mouse macrophage-like cells (ATCC TIB-71™) were grown in Dulbecco modified Eagle medium (DMEM) supplemented with 10% FBS, penicillin and streptomycin at 37°C, 5% CO2. For routine maintenance, RAW 264.7 cells were grown to 80% confluence and sub-cultured every two days.

### Lipoprotein Isolation and Oxidation

LDL (*d* = 1.019 to 1.063 g/ml) was isolated from plasma of donors who were free from clinically apparent disease, and oxidatively modified using Cu_2_^+^ as described previously [46], [50], [51]. The degree of LDL oxidation was monitored continuously by fluorescence emission at 234 nm using a fluorescence spectrophotometer (SLM-AMINCO® Series 2, Spectronic Instruments, Rochester, NY) and stopped when the fluorescence values reached the peak (≥1.1 fluorescence units). The oxidative modification of LDL was evaluated by quantitation of conjugated dienes [50], [51]. LDL modification was verified by particle migration on the Paragon® Electrophoresis System (Beckman Coulter, Fullerton, CA). Briefly, lipoproteins (5 µg protein each) were loaded onto the gel and subjected to electrophoresis for 30 min at 100 V. Particle modification was verified by migration of the oxLDL relative to native LDL (N-LDL) samples.

### Preparation of oxLDL-IC Immune Complexes

Immune complexes containing oxLDL were prepared with human oxLDL and human anti-oxLDL antibodies as described previously [2], [9], [46]. Keyhole limpet hemocyanin immune complexes (KLH-IC) were used as control immune complexes because KLH has a molecular weight comparable to LDL and because it can engage Fcγ receptors similar to oxLDL-IC but does not contain lipoproteins. KLH-IC were also prepared as described previously [9], [46]. After precipitation, immune complexes were re-suspended in Dulbecco's phosphate buffered saline (DPBS) and the concentrations of total protein were determined using the BCA protein assay (Pierce, Thermo Scientific, Rockford, IL). Labeled oxLDL-IC were prepared with fluorescently labeled oxLDL.

### Labeling of oxLDL with Lipophilic Fluorescent Dyes

Fluorescent labeling of the lipid moiety of oxLDL was performed as described previously [52] with modification. Briefly, oxLDL (1.0 ml, 1.0 mg protein) was mixed with lipoprotein-deficient serum (LPDS, 1.0 ml), and then filter (0.22 µm) sterilized. A 50-µl aliquot of DiI:1,1′-dioctadecyl-3,3,3′,3-tetramethylindocarbocyanine perchlorate (DiI) or DiO:3,3′-dioctadecyloxacarbocyanine perchlorate (DiO) (Invitrogen, Carlsbad, CA), each 3.0 mg/ml in DMSO (Sigma, St. Louis, MO), was added to the oxLDL-LPDS mixture. The mixture was gently mixed and incubated at 37°C for 8 h. To isolate the labeled oxLDL, the density of the solution containing the fluorescent labeled LDL was raised to 1.225 g/ml with solid KBr, and the solution loaded into a polymer ultracentrifuge tube (13-ml tube, Beckman). Tubes were then filled with a saline solution whose density was adjusted to 1.21 g/ml with solid KBr. The labeled LDL was then floated out of the mixture by ultracentrifugation with a Beckman SW41 Ti rotor at 41,000 rpm, 36 h, 4°C. The labeled oxLDL floating at the top of the tube was aspirated and the density of this solution was raised to 1.225 g/ml and the solution was again centrifuged at 41,000 rpm, 36 h, 4°C. The top layer was aspirated, dialyzed against NaCl-EDTA solution (150 mM NaCl/300 µM EDTA), pH 8.6) then filter-sterilized and stored at 4°C. The concentration of protein in the labeled oxLDL was determined using the BCA protein assay, and the differential electrophoretic mobility of labeled oxLDL in agarose was determined as described above.

### Fluorescent Labeling of the Protein Moiety of oxLDL

Fluorescent labeling of the protein moiety of oxLDL was performed using Alexa Fluor® 546 carboxylic acid, succinimidyl ester (Invitrogen) (Alexa 546) according to manufacturer's protocol. Briefly, oxLDL (2 mg protein) was dialyzed in 0.1 M NaHCO_3_ buffer, pH 8.3. Alexa 546 (1 mg/100 µL DMSO) was then added to oxLDL solution (25 µL:2 ml, respectively). This solution was incubated for 1 h at room temperature in the dark. The protein-dye conjugate was separated from free unbound dye by PD-10 column. The sample was eluted using PBS; 0.5-ml fractions were collected. The peak fractions were pooled, dialyzed against NaCl-EDTA solution, and then filter-sterilized and stored at 4°C. Protein concentration was determined using BCA protein assay and differential migration of labeled oxLDL was determined as described above.

### Fluorescence Activated Cell Sorting (FACS)

Internalization of fluorescently labeled oxLDL was evaluated by flow cytometry. Cells (8×10^5^ cells/ml) were serum starved and IFN-γ treated as described above. Cells were then treated with DiO-oxLDL, DiI-oxLDL, or Alexa 546-oxLDL (5, 10, 25, or 50 µg/ml) for 90 min. Cells were then washed three times with DPBS, with propidium iodide added to the last wash for dead cell exclusion. Cells were then analyzed by fluorescence activated cell sorting with a Becton Dickinson FACSAria Cell Sorter (BD Bioscience, San Jose, CA).

### Expression of GFP-tagged human HSP70 and HSP70B' in RAW 264.7 cells

HSP70-GFP and HSP70B'-GFP expression vectors were prepared and inserted into RAW 264.7 cells according to the protocol described in our recent study [16].

### Fluorescent Labeling of Transferrin for Tracking of Labeled Lipoprotein Moieties in Endosomal Compartment

Transferrin was labeled using Alexa Fluor® 488 Protein Labeling Kit or Alexa Fluor® 633 Protein Labeling Kit (Invitrogen) according to manufacturer's protocol. The degree of labeling was calculated and found to be 3.11 mole of Alexa Fluor® dye per mole of transferrin. U937 cells (1×10^6^ cells/ml) were simultaneously treated with Alexa 488-transferrin (5 µg/ml) and with either DiI-labeled lipid moiety or Alexa 546-labeled protein moiety of oxLDL and oxLDL-IC for 90 min and 5 h. Cells were treated with labeled oxLDL (24 µg/ml) or labeled oxLDL-IC (32 µg/ml). Higher protein concentration of oxLDL-IC was used as compared to oxLDL to normalize the amount of oxLDL administered to the cells. Cells were then pelleted, fixed with 4% formaldehyde, then washed three times with DPBS. Cells were then suspended in 100 µl DPBS and 10-µl aliquots were loaded in 10-µl glass capillaries (Idaho Technology, Salt Lake City, UT) as described previously [53]. The capillaries were sealed and cells visualized using confocal microscopy (Zeiss LSM 510 Meta Laser Scanning Confocal Microscope, Carl Zeiss MicroImaging, Inc., Thornwood, NY).

RAW 264.7 cells transfected with HSP70-GFP or HSP70B'-GFP were plated in sterile glass bottom 96-well plates (MatTek Corporation; Ashland, MA), then treated with DiI-oxLDL (24 µg/ml) or DiI-oxLDL-IC (32 µg/ml) in serum-free DMEM for 3 h at 37°C, 5% CO2. Alexa 633-transferrin (10 µg/ml) was then added for an additional 2 h. Cells were then fixed with 4% formaldehyde, washed three times with DPBS, and visualized using confocal microscopy.

### Tracking of Labeled Lipoprotein Moieties in Lysosomal Compartment

U937 cells were treated with labeled oxLDL (18 µg/ml) or labeled oxLDL-IC (24 µg/ml) for 90 min and 5 h. Cells were treated with 50 nM Lyso Tracker Green DND-26 (Invitrogen) (Lyso Tracker-26) 30 min prior to conclusion of incubation time with labeled oxLDL and oxLDL-IC. Live cells were then washed three times in serum free IMDM, suspended in 100 µl IMDM, and 10-µl aliquots were loaded in capillaries and sealed as described above. The live cells were visualized using Zeiss LSM 510 Meta Confocal Microscope.

### Tracking of Labeled Lipoprotein Moieties of oxLDL and oxLDL-IC Administered Simultaneously or Sequentially

U937 cells were treated with DiO-oxLDL (24 µg/ml) and DiI-oxLDL-IC (32 µg/ml) in parallel for 5 h. In comparison studies, cells were incubated sequentially with DiI-oxLDL-IC for 2 h followed by DiO-oxLDL for an additional 3 h. To determine if the parallel or sequential incubation of labeled lipoproteins resulted in localization of the fluorescent labels within the lysosomal compartment, Lyso Tracker Blue DND-22 (Invitrogen) (Lyso Tracker-22) was used. This tracker is a blue fluorescent dye that stains acidic compartment in live cells with excitation/emission maxima 373/422 nm. U937 cells were treated with 50 nM of Lyso Tracker 30 min prior to conclusion of incubation time with labeled oxLDL and oxLDL-IC. Live cells were then washed three times in serum free IMDM, suspended in 100 µl IMDM, and 10-µl aliquots were loaded into capillaries and sealed as described above. The live cells were visualized using a Zeiss LSM 510 Meta Confocal Microscope.

### Detection of Mitochondrial Membrane Potential and Changes in ROS and RNS

MitoTracker® Deep Red FM (Invitrogen) (1 mM in DMSO) was used to probe the mitochondria. This dye passively diffuses across the plasma membrane and accumulates in active mitochondria, dependent upon membrane potential. For detection of ROS, 5-(and-6)-chloromethyl-2′,7′-dichlorodihydrofluorescein diacetate, acetyl ester (CM-H2DCFDA) (Invitrogen) was used to detect the generated intracellular H_2_O_2_. CM-H_2_DCFDA is a cell-permeant indicator that is not fluorescent until removal of the acetate groups by intracellular esterases and oxidation occurs. CM-H_2_DCFDA (5 mM) was prepared in DMSO according to manufacturer's protocol. U937 cells (4×10^5^ cells/ml) were incubated in phenol red-free IMDM supplemented with 10% fetal bovine serum, 100 units/ml penicillin, 50 µg/ml streptomycin, and IFN-γ (200 ng/ml) for 18 h prior to addition of experimental treatments. Cells were treated with oxLDL (90 µg/ml), oxLDL-IC, KLH-IC (120 µg/ml) in IMDM for 30 min, 90 min, and 5 h. Cells were treated with CM-H_2_DCFDA (5 µM), and Mito Tracker (100 nM) for 30 and 15 min, respectively, prior to conclusion of incubation time with treatments. Cells were then fixed and visualized using confocal microscopy.

For detection of RNS, 4-amino-5-methylamino-2′,7′-diflurofluorescein diacetate (Invitrogen) was used to detect the generated intracellular nitric oxide (NO). DAF-FM diacetate is not fluorescent until it is de-acetylated by intracellular esterases and reacts with NO to form a fluorescent benzotrizole. DAF-FM diacetate (5 mM) was prepared in DMSO according to manufacturer's protocol. U937 cells were incubated with L-arginine (100 µM) in DPBS and DAF-FM diacetate (10 µM) for 1 h, and then cells were washed two times with serum and phenol red-free IMDM. Cells were treated with oxLDL (90 µg/ml), oxLDL-IC, KLH-IC (120 µg/ml) in IMDM for 30 min, 90 min, and 5 h. Fifteen minutes prior to conclusion of incubation time with treatments cells were treated with Mito Tracker (100 nM). Live cells were then washed twice with IMDM visualized using confocal microscopy.

### Ethics Statement

Human LDL and antibodies were obtained from healthy volunteers. Informed written consent as approved by the Institutional Review Board for Human Research of the Medical University of South Carolina was obtained from each donor included in this study.
